# Plastic-Colonizing Fungi in the Eastern Mediterranean Sea: Community Structure and Physiological and Transcriptional Responses of *Aspergillus* to Pristine and Weathered PET

**DOI:** 10.3390/ijms27156861

**Published:** 2026-07-30

**Authors:** Sheli Shlomo Itzahri, Denis Popov, Keren Davidov, Matan Oren

**Affiliations:** School of Biomedical Sciences, Ariel University, Science Park, Ariel 40700, Israel; shlomoi@ariel.ac.il (S.S.I.); popov.denis98@gmail.com (D.P.); kerendav@ariel.ac.il (K.D.)

**Keywords:** plastisphere, marine fungi, plastic-associated mycobiome, *Aspergillus niger*, culture-dependent bias, polyethylene terephthalate, UV-weathered PET, peroxidase activity, fungal attachment, plastic biodeterioration, Eastern Mediterranean Sea

## Abstract

Marine plastic debris provides persistent artificial substrates for microbial colonization, yet the fungal component of the plastisphere remains poorly characterized. Using ITS metabarcoding and culture-based isolation approaches, we characterized the early mycobiome associated with polyethylene (PE), polypropylene (PP), polystyrene (PS), and polyethylene terephthalate (PET) plastic pellets deployed in an Eastern Mediterranean Sea (EMS) marina. The sequence-based communities were taxonomically heterogeneous, with only a limited effect of polymer type. Ascomycota, Zoopagomycota, and Basidiomycota were the dominant phyla identified by the ITS sequence analysis, while *Linderina* and *Starmerella* were the dominant genera. In contrast, the cultured isolates belonged exclusively to the phyla Ascomycota and Mucoromycota, with the *Aspergillus* genus accounting for 52.3% of the isolates. We further recorded physiological responses of 11 *Aspergillus* isolates to pristine and UV-weathered PET, including attachment, pigmentation, and plastic gravimetric weight loss. *A. niger* OC26 also exhibited strong peroxidase activity, together with a broad transcriptional response to PET exposure, including 216 upregulated genes and significant enrichment of peroxidase- and membrane-associated functions. UV weathering induced a limited but distinct additional transcriptional shift. Together, these findings indicate that early plastic-associated fungal communities in the EMS are taxonomically diverse and include metabolically versatile *Aspergillus* strains that mount distinct physiological and transcriptional responses to PET exposure.

## 1. Introduction

Since the surge in plastic use ~70 years ago, mismanaged plastic waste has accumulated in the ocean, often exceeding the volume of natural floating debris. An estimated 19 to 23 million metric tons of plastic enter all aquatic systems annually [1], while current estimates suggest that 82–358 trillion plastic particles are afloat in the world’s oceans [2]. Unlike natural floating substrates such as wood or macroalgae, petroleum-based polymers like polyethylene (PE), polypropylene (PP), polyethylene terephthalate (PET) and polystyrene (PS) are highly durable and are estimated to take hundreds and even thousands of years to fully break down [3,4].

Consequently, marine plastic debris provides a stable anthropogenic habitat that is rapidly colonized by diverse microbial communities, forming a distinct marine ecosystem referred to as the “plastisphere” [5]. The recent accumulation of plastics in the marine environment has introduced a novel, persistent, and chemically distinct ecological niche that may impose new selective pressures on marine microorganisms. Hence, plastisphere communities are not merely passive assemblages of surface-attached cells, but often contain microorganisms with traits that reflect adaptation to life on plastic surfaces, including enhanced attachment, biofilm formation, utilization of plastic additives or weathering-derived compounds, and the expression of enzymes potentially involved in polymer transformation [6,7,8]. Accordingly, it was shown that the early marine microbial communities that are formed on newly introduced plastic surfaces are enriched with polymer-specific pioneer colonizers compared to later biofilm development stages for which the polymer surface is no longer exposed [9].

The plastisphere communities exhibit unique taxonomic, functional, and structural characteristics compared with their surrounding planktonic counterparts and often span multiple trophic levels, including producers, consumers, predators, and decomposers [5]. These communities are highly heterogeneous and are shaped by the spatiotemporal conditions including temperature, salinity, pH, dissolved gases and light availability [10,11]. Weathering of plastics is another major factor in microbial colonization [12,13,14]. UV-induced photo-oxidation of floating plastic polymers introduces oxygen-containing functional groups, reduces hydrophobicity, increases surface roughness, and generates cracks and oxidized low-molecular-weight compounds, thereby modifying the cues that govern microbial attachment and interaction with plastic surfaces [14,15].

Among the abundant eukaryotes in the marine plastisphere are fungi, yet their ecological and functional roles on marine plastics remain significantly less explored than those of bacteria [16,17,18]. Fungi are heterotrophic eukaryotes characterized by diverse morphologies, life histories, and ecological strategies. Many function as saprotrophs, secreting extracellular enzymes, including laccases and peroxidases, that evolved to degrade natural organic biopolymers such as lignin and cellulose [19]. The resulting low-molecular-weight products are then absorbed and assimilated by the fungal cells. Depending on the substrate and environmental conditions, this process can result in complete biomineralization, reflected by CO_2_ release, or partial degradation into smaller carbon compounds that may subsequently be consumed by other microorganisms [20]. Filamentous fungi further enhance surface-associated degradation through hyphal growth, which allows them to explore and physically penetrate surfaces, anchor biofilms, and concentrate enzymatic activity at the substrate interface [21]. Synthetic polymers, such as PE and PET, contain recalcitrant carbon-rich or ester-containing structures that can resemble natural polymeric substrates in their resistance to degradation. For these reasons, marine fungi are increasingly recognized as potential agents of plastic biodeterioration, transformation, and, in some cases, biomineralization [22,23,24].

Although marine plastic-associated fungal taxonomy may greatly vary across different geographic locations and environmental conditions, the dominant plastisphere fungal phylum is Ascomycota in most cases [25,26]. Specific genera within this phylum, such as *Aspergillus* and *Trichoderma*, have already demonstrated the ability to utilize polymers like PE as a carbon source through enzymatic activity [27,28,29]. However, little is known about the Eastern Mediterranean Sea (EMS) plastisphere mycobiome and the physiological responses and interactions of its members with plastic surfaces.

In this study, we investigated the diversity and functional potential of pioneer fungal colonizers associated with PE, PP, PET, and PS plastic polymer pellets deployed in an EMS marina. We used ITS metabarcoding to characterize polymer-associated fungal communities, complemented by a culture-dependent approach for the morphological and functional characterization of fungi isolated from marine plastics. We further examined the responses of 11 culturable *Aspergillus* isolates to pristine PET (PPET) and UV-weathered PET (WPET) by assessing adhesion, pigmentation, peroxidase activity, and potential plastic biodegradation. To explore the molecular basis of these responses, we investigated a highly active *Aspergillus niger* isolate culture under three conditions: without plastic and in the presence of either PPET or WPET. This approach enabled us to identify differentially expressed genes and pathways potentially involved in fungal adhesion to PET, oxidative stress responses, pigmentation and plastic-associated degradation processes in this isolate.

## 2. Results

### 2.1. Diversity of Plastic-Derived Fungi from an EMS Marina

To characterize the fungal community associated with marine plastics in the EMS, we performed ITS metabarcoding using DNA extracted from PE, PP, PS, and PET beads submerged in Herzliya Marina for two weeks. The amplified ITS2 region was sequenced using the Oxford Nanopore MinION platform. The sequencing run yielded approximately 25.44 million high-quality, basecalled reads, corresponding to an average of 1.06 million reads per sample. Most reads fell within the expected length range of approximately 300–500 bp (Figure S1).

The processed ITS reads were analyzed to assess alpha diversity within samples and beta diversity among samples. Fungal richness, estimated using the Chao1 index, did not differ significantly among polymer types (Figure S2a). In contrast, Shannon diversity showed modest but significant variation across polymers (Figure S2b; Kruskal–Wallis test, *p* = 0.027), with PP samples displaying higher diversity values on average. Bray–Curtis dissimilarity analysis did not reveal strong clustering according to polymer type. Nevertheless, PERMANOVA detected a significant compositional difference between PE and PP communities (FDR = 0.048; Figure S2c). Overall, these results indicate that polymer type exerted only a limited influence on the structure of the two-week-old pioneer mycobiome.

**Figure 1:**
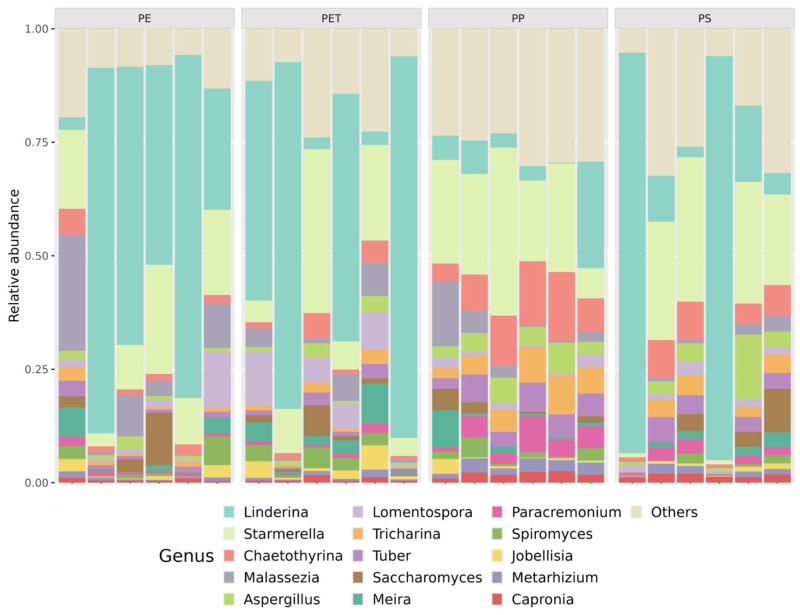
Relative abundance of the 15 most dominant fungal genera identified in each of the plastic polymer samples (six repeats per polymer).

Variations were observed in the relative abundance of the dominant genera among polymers and biological replicates (Figure 1). Overall, *Linderina* was the most abundant genus, showing the highest relative abundance in half of the samples (12/24), primarily in PE and PET, accounting for an average of 33.9% of ITS reads per sample. Most of the remaining samples were dominated by the yeast genus *Starmerella*, particularly in PP and PS, with an average relative abundance of 16.73%. Other genera represented within the 15 most abundant taxa included *Chaetothyrina* (4.33%), *Malassezia* (4.18%)*, Lomentospora* (2.78%), *Trichanina* (2.63%), *Tuber* (2.62%), *Aspergillus* (2.33%), *Saccharomyces* (2.22%), *Meira* (2.15%), *Paracremonium* (1.87%), *Spiromyces* (1.67%), *Capronia* (1.23%), *Jobellisia* (1.18%), and *Metarhizium* (1.17%).

### 2.2. Isolation of Pure Marine Fungal Strains from Plastic

Following inoculation of the recovered plastic pellets and glass beads onto agar plates, visible hyphal growth typically appeared within 48 h to a week at 28 °C (Figure S3), whereas full mycelial expansion across the agar surface (Figure S4) required up to two weeks. Our inoculation procedure yielded consistent fungal growth from the samples onto malt extract agar (MEA) medium, most often as a single strain per pellet (Figure S3). In total, 109 isolates representing 21 distinct morphotypes grew out of the inoculated samples, with only two isolates obtained from the glass beads (Table S1). More than half of the isolates were recurrently isolated from at least two different plastic polymers (Table S1). The most frequently recovered isolate was OC26 (26 of the 109 occurrences), with the closest database match to *Aspergillus niger*, followed by OC2 (23 occurrences), identified as *Cladosporium sphaerospermum*. Other common isolates included OC9 (*Alternaria destruens*; 11 occurrences), OC15 (*Waltergamsia pilosa*; 8 occurrences), OC8 (*Allophoma yuccae*; 6 occurrences), OC11 and OC3 (*Penicillium steckii* and *Mucor* sp. respectively; 5 occurrences each) (Table 1).

### 2.3. Fungal Isolation Introduced Taxonomic Bias Toward Ascomycota and the Genus Aspergillus

**Figure 2:**
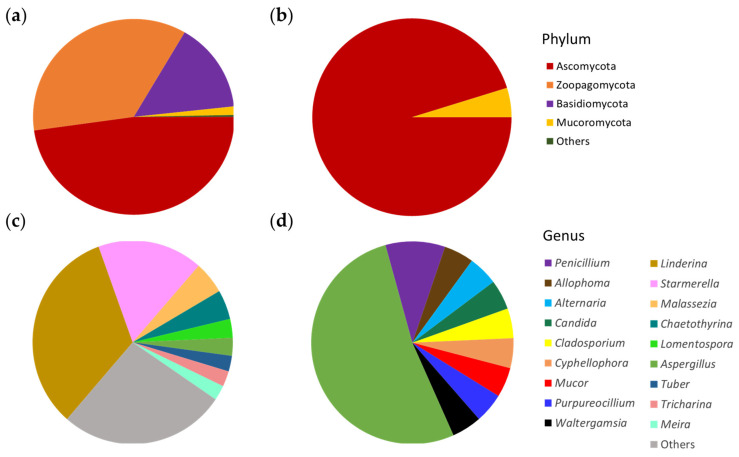
Taxonomic composition of fungal communities associated with marine plastic samples. (**a**,**b**) Plastic-associated phylum-level fungal composition based on ITS metabarcoding (**a**) or isolation on malt extract agar (MEA) medium (**b**). (**c**,**d**). Fungal genus-level composition based on ITS metabarcoding (**c**) or isolation on MEA medium (**d**).

ITS metabarcoding detected fungi from several phyla, predominantly Ascomycota, Zoopagomycota, Basidiomycota, and Mucoromycota (Figure 2a), whereas the cultured isolates were restricted to only two phyla: Ascomycota and Mucoromycota (Figure 2b). Ascomycota was strongly overrepresented in the cultivation-derived dataset, comprising 95.2% of all isolates compared with only 47.8% of the ITS metabarcoding dataset. This bias was even more pronounced at the genus level. The composition of the ten most abundant genera differed substantially between the metabarcoding dataset and the cultivated isolate collection. Except for *Aspergillus*, no genera were shared among the ten most abundant genera in both datasets. The metabarcoding dataset was dominated by *Linderina* (33%) and *Starmerella* (17%), followed by *Malassezia* (5.2%) and *Chaetothyrina* (4.7%). In contrast, the cultivated collection was dominated by *Aspergillus*, which accounted for 52.3% of all isolates, followed by *Penicillium* (9.5%).

### 2.4. Plastic-Associated Aspergillus Isolates

**Figure 3:**
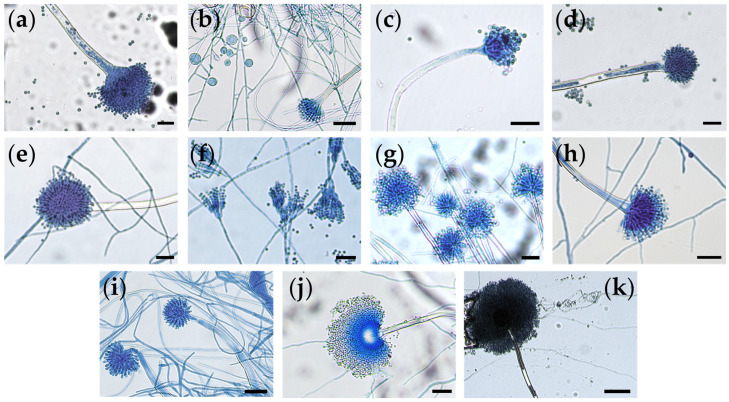
Fruiting bodies of different *Aspergillus* isolates stained with Lactophenol Cotton Blue (LPCB): (**a**) OC6; (**b**) OC7; (**c**) OC10.2; (**d**) OC12; (**e**) OC14; (**f**) OC18; (**g**) OC20; (**h**) OC21; (**i**) OC23; (**j**) OC24; (**k**) OC26. Scale—20 µm.

Because *Aspergillus* represented a substantial proportion of the plastic-derived fungal isolate collection, this genus was selected for further investigation of fungal-plastic interactions. In total, 11 distinct *Aspergillus* isolates were recovered, differing in colony morphology and pigmentation (Figure S4), as well as in the size, internal structure, and spore-bearing capacity of their fruiting bodies (Figure 3). The morphological characterization combined with the ITS barcoding results suggested that each isolate belonged to a distinct *Aspergillus* strain. However, the final genus- and species-level identities were established based on the ITS2 sequence (Table 1).

### 2.5. Response of Aspergillus Isolates to the Presence of Pristine PET (PPET) and Weathered PET (WPET)

**Figure 4:**
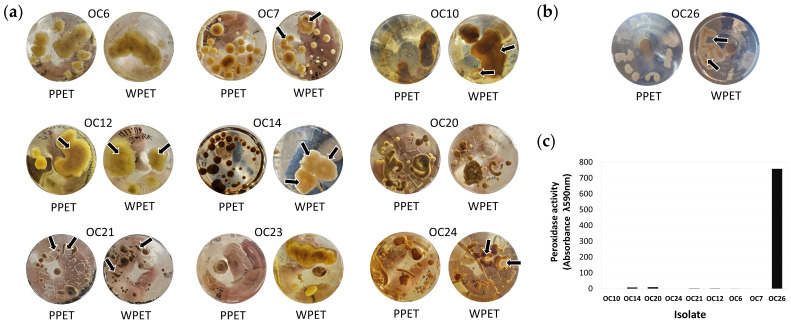
*Aspergillus* responses to the presence of PET. (**a**) Differences in pigmentation and attachment responses of *Aspergillus* isolates to PPET versus WPET. (**b**) Isolate OC26 in the presence of PPET versus WPET. (**c**) Normalized peroxidase activity in the presence of WPET, measured by the chromogenic reaction produced using the Amplex™ Red Hydrogen Peroxide/Peroxidase Assay Kit (Invitrogen, Thermo Fisher Scientific, Carlsbad, CA, USA). Attachment to the square PET surfaces is indicated by black arrows.

The different Aspergillus isolates showed physiological response to the presence of the PET sheets. Responses included pigmentation of dark brownish color, surface attachment in some cases, and peroxidase activity. The increased pigmentation occurred in the presence of PPET (isolates OC14, OC12) or WPET (isolates OC23 and OC26) or in both (isolates OC6, OC7, OC10.2, OC20, OC21, and OC24) (Table 2, Figure 4a,b). Surface attachment under gentle shaking was observed in isolates OC7, OC10.2, OC12, OC14, OC21, OC24, and OC26, and in most cases occurred exclusively on WPET (Table 2). Fungal hyphae frequently accumulated along the thin edges of the WPET squares (Figure 4a,b). Significantly high peroxidase activity was detected in *A. niger* cultures in the presence of WPET, while the other *Aspergillus* isolates presented either very little or no activity in one-week cultures (Table 2, Figure 4c). The six-week (42-day) gravimetric weight-loss assay revealed a 0.10–0.21% reduction in WPET weight relative to the corresponding WPET controls in 7 of the 10 tested strains (Table 2).

### 2.6. Differential Trasncriptomic Responses of A. niger OC26 to WPET and PPET

**Figure 5:**
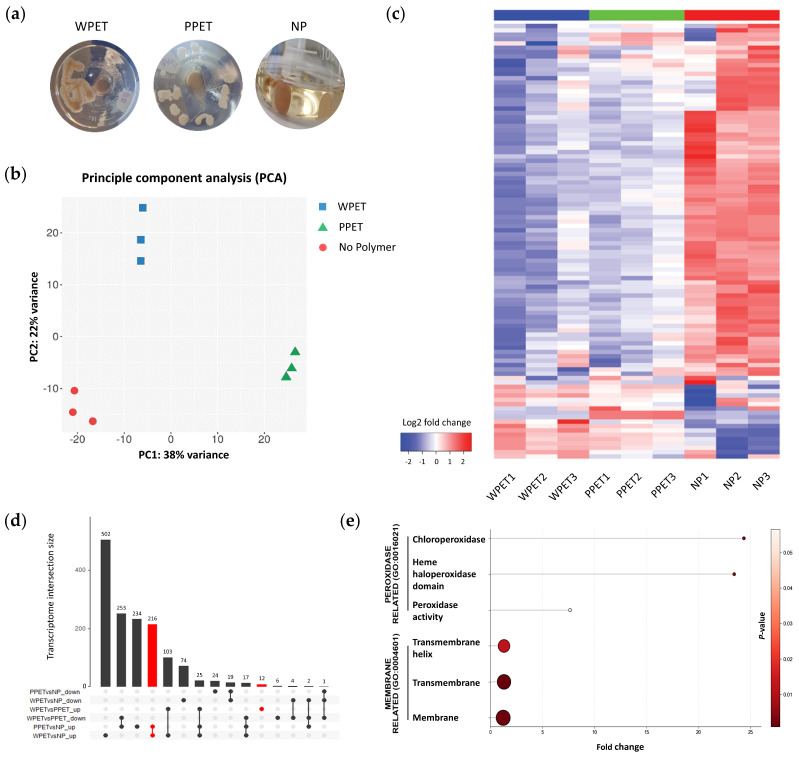
Transcriptional response of fungal isolate OC26 to the presence of WPET and PPET. (**a**) OC26 cultures in the presence of PPET, WPET and without plastic (NP). (**b**) Principal component analysis (PCA) of the three conditional transcriptomes (with three repeats each). (**c**) Heatmap of the top differentially expressed genes with Log2FC > 2 and *p*-value < 0.05 across WPET, PPET, and NP. (**d**) Numbers of differentially expressed genes according to Deseq2 DEG. In red—intersections of genes that were upregulated in the presence of PET (PPET + WPET, 216 genes) compared with no-plastic control, or in the presence of WPET compared to PPET (12 genes). (**e**) Top enrichment clusters of genes upregulated in response to the presence of plastic (WPET and PPET), identified using the DAVID platform.

mRNA sequencing was performed on *A. niger* OC26 cultures grown either in the absence of PET (NP) or in the presence of PPET or WPET (Figure 5a; *n* = 3 per condition). Sequencing generated a total of 11.22 million reads, with read lengths ranging from 100 bp to 3.4 kb and an N50 read length of 1.15 kb (Figure S5). Of these, 9,622,211 reads (85.74%) mapped to the reference *A. niger* genome (GCF_000002855.4), corresponding to 10,828 expressed genes.

Principal Component Analysis (PCA) revealed clear separation of samples according to the three experimental conditions (Figure 5b), indicating a condition-dependent transcriptomic response. Consistently, analysis of the 120 most variable genes showed marked transcriptional differences between PET-exposed cultures (PPET and WPET), compared to the NP cultures (Figure 5c). Differential expression analysis identified 216 genes that were overexpressed in PET-containing cultures relative to NP cultures (Table S6), whereas 12 genes were overexpressed in WPET cultures compared with PPET cultures (Figure 5d). Comparative GO-term enrichment analysis revealed strong enrichment of peroxidase-related functions, with fold changes ranging from 6.8 to 24.4, followed by more moderate but significant enrichment of membrane-related functions, with fold changes ranging from 1.2 to 1.5 (Figure 5e).

Among the differentially expressed genes (Table S6), promising candidates were identified that may contribute to the physiological adaptation of *Aspergillus niger* to the polymer surface and the metabolism of PET-associated compounds. These included genes encoding a peroxidase [An02g10480], cytochrome P450 [An11g08810], dienelactone hydrolase [An14g01430], aromatic ring-opening dioxygenase LigB subunit [An12g07530], salicylate hydroxylase [An07g00550], 2,4-dichlorophenol 6-monooxygenase [An04g04680], and several oxidoreductases [An04g08020, An04g04170, An02g10680, and An09g01080], which may contribute to oxidative stress management and the transformation of aromatic intermediates. The induction of an adhesin [An16g04900], a FAS1 domain-containing protein [An16g07370], MFS transporters [An16g01000, An04g08250, An07g01220, An02g08970, An01g04100, An12g03550, and An15g02190], an ABC transporter [An09g01700], catalase R [An01g01550], and oxidation resistance protein 1 [An16g02160] further suggests coordinated surface attachment, detoxification, and stress adaptation. In addition, the induction of a polyketide synthase [An04g04340] and a Nor-1-like ketoreductase [An11g02460] may indicate activation of pigment-associated secondary metabolism, potentially contributing to melanization and protection against PET-associated oxidative stress. Nevertheless, the direct involvement of these genes in melanin production or PET depolymerization remains to be experimentally validated.

## 3. Discussion

In this study, we applied an integrative approach to characterize the early fungal colonization of marine plastics in the EMS and to investigate the physiological and molecular responses of plastic-associated fungi to plastic exposure. By combining ITS metabarcoding, culture-dependent isolation, physiological assays, and transcriptomic profiling of an *A. niger* isolate, we linked community-level patterns with functional insights into fungal adaptation to and interaction with plastic surfaces.

The plastic-attached fungal composition was partially consistent with previous marine plastisphere studies showing that plastic-attached fungal communities are commonly dominated by Ascomycota, with additional contributions from Basidiomycota and Mucoromycota or other early-diverging fungal lineages [20,30,31,32]. Chytridiomycota, a primarily aquatic phylum that is frequently reported in marine plastisphere fungal communities, did not represent a significant fraction of the fungal community in our study [30]. This suggests that chytrid-mediated parasitic or host-associated interactions were not a prominent feature of the early plastisphere mycobiome under the conditions examined. In contrast, Zoopagomycota (Formerly part of the Zygomycota phylum) were notably represented in our dataset, suggesting potential trophic complexity within the early plastisphere mycobiome. As members of this phylum often exhibit parasitic, predatory, or host-associated lifestyles [33], their presence may reflect biotic interactions within the biofilm, although this cannot be inferred from metabarcoding data alone.

At the genus level, several dominant taxa, including *Linderina*, *Starmerella*, *Malassezia*, *Saccharomyces*, and *Tuber*, are rarely reported as major components of plastisphere fungal communities, which more commonly include *Cladosporium*, *Alternaria*, *Aspergillus*, and *Penicillium* [30,31,32,33,34]. Their abundance may reflect opportunistic colonization by locally available terrestrial, animal-associated, or airborne fungi during the short 14-day deployment rather than specific adaptation to plastic surfaces. Because these taxa were identified only by ITS metabarcoding and were not recovered in culture, some detections may represent transient or non-viable propagules. Nevertheless, their consistent occurrence across replicates suggests that they are genuine components of the early EMS plastisphere mycobiome.

We note that the composition of the plastisphere mycobiome is strongly influenced by the geographic location, the local environmental conditions, and the duration of plastic submersion [34,35]. Accordingly, a consistent and broadly shared taxonomic core is not necessarily expected across different studies. The Mediterranean Sea is a semi-enclosed, highly oligotrophic basin characterized by high solar irradiance and strong seasonal temperature gradients [36,37]. In addition to these regional environmental pressures, the Herzliya Marina represents a locally impacted coastal habitat, exposed to petroleum-derived hydrocarbons such as boat fuel, lubricating oils, and other marina-associated contaminants. Its potential to harbor fungi adapted to hydrocarbon-rich conditions was therefore a key reason for selecting this site. Nevertheless, differences among studies are expected even when they are conducted at the same geographic location. Indeed, our previous mycobiome analysis conducted in Herzliya Marina revealed a different composition of plastisphere-associated fungal genera [38]. These differences may reflect the differences in the year and season of deployment, as well as in the duration of plastic incubation between the two studies.

Our plastic mycobiome analysis suggests that pioneer fungal colonization of marine plastics is only weakly shaped by polymer-specific selection and may instead be influenced by other niche-specific ecological factors or stochastic events. Similar results were obtained in a two-weeks brackish ecosystem incubation study by [30], showing no significant differences in fungal 18S community composition on PE compared to PS surfaces. A more recent marina incubation study by [32] that tested the fungal community on conventional PS foams and their biodegradable alternatives over a period of nine weeks, showed a limited, context-dependent polymer-specific effect.

In addition to the ITS metabarcoding, culture-independent approach, we utilized traditional cultivation techniques to isolate live fungi. While metabarcoding provides a broader view of environmental fungal diversity, including uncultured or low-abundance taxa, culture-based approaches selectively recover viable fungi capable of growing under the imposed laboratory conditions [16]. As expected, laboratory cultivation recovered a narrower mycobiome fraction, strongly biased toward Ascomycota and particularly the genus *Aspergillus*, which accounted for more than half of the isolate collection. The discrepancy between our culture-dependent isolation and the ITS metabarcoding results is consistent with previous marine fungal studies showing that these approaches capture partly overlapping but distinct fractions of fungal diversity [20,39].

Recent literature highlights several examples of marine and coastal fungi with plastic-associated degradative potential. The marine fungus *Zalerion maritimum* was reported to cause substantial mass loss of PE microplastics over a two-week incubation period [40], while other ascomycete genera, including *Fusarium* and *Penicillium*, have also shown polymer-modifying capabilities [22,41]. For example, *Fusarium solani* has been shown to hydrolyze PET into terephthalic acid (TPA) through cutinase activity [23], and several *Penicillium* species isolated from marine or coastal environments have demonstrated the ability to modify or degrade PE and high-density polyethylene (HDPE) [42,43]. Members of the genus *Aspergillus* are among the most frequently reported fungi associated with plastic colonization and biodegradation, reflecting their ecological flexibility, stress tolerance, and broad enzymatic repertoire [22,44]. Marine-adapted strains of *Aspergillus*, including *A. terreus*, *A. flavus*, and *A. niger*, have been reported to attach to and modify synthetic polymers such as PE, polyurethane (PU), and PET. These interactions involve mechanisms such as hyphal colonization, surface erosion, oxidative activity, and the secretion of extracellular enzymes, including esterases, lipases, cutinases, laccases, and peroxidases [22,45]. However, despite these observations, the broader physiological and transcriptional responses of *Aspergillus* to plastic exposure remain poorly understood.

In this study, we demonstrated pronounced physiological responses of *Aspergillus* isolates to PET, including surface attachment, morphological changes, and altered pigmentation. Most isolates exhibited attachment to PET surfaces, with attachment generally more pronounced on WPET. Interestingly, several isolates also displayed phenotypic changes in the presence of PET despite no detectable surface attachment, including alterations in colony morphology and pigmentation. These observations suggest that *Aspergillus* may possess sensitive mechanisms for detecting and responding to PET or PET-associated chemical cues without requiring direct attachment. Notably, pigmentation responses were not consistently associated with either attachment capacity or UV weathering, as most isolates exhibited stronger pigmentation in the presence of pristine PET.

The gravimetric assay revealed only limited WPET weight loss (0.1–0.21%, corresponding to approximately 0.002–0.005% per day). Given the low magnitude of mass loss, the gravimetric results should be interpreted as suggestive rather than definitive evidence of PET degradation. Direct confirmation of polymer modification requires further use of complementary approaches, such as FTIR spectroscopy and SEM. We therefore interpret the recovered *Aspergillus* isolates not as specialized or efficient PET degraders, but rather as metabolically versatile colonizers capable of sensing, attaching to, and potentially modifying weathered plastic surfaces during early-stage colonization. The detection of peroxidase activity in *A. niger* OC26 provides functional support for the involvement of oxidative processes in plastic biodeterioration. Fungal peroxidases, together with laccases and other oxidoreductases, are involved in the modification of recalcitrant organic substrates and have been implicated in the oxidative transformation of synthetic polymers [22,46]. Accordingly, the heightened peroxidase activity detected in *A. niger* OC26 cultures exposed to WPET indicates a distinct enzymatic response to PET exposure. This physiological response was further supported by the transcriptomic data, which revealed strong enrichment of peroxidase-related functions, a directly upregulated peroxidase candidate, and multiple genes potentially involved in activating a broader oxidative system responsible for both the generation and detoxification of reactive oxygen species during PET exposure. Collectively, these findings suggest that *A. niger* OC26 may actively contribute to oxidative modification of the plastic surface and/or the metabolism of weathering-derived leachates.

*A. niger* OC26, similar to several of the other *Aspergillus* isolates, exhibited increased pigmentation in the presence of WPET. This response may reflect stress-induced melanization triggered by the oxidized plastic interface, potentially protecting fungal hyphae against reactive oxygen species (ROS) associated with the polymer surface [15,47,48,49]. Consistent with this interpretation, our transcriptomic analysis revealed the induction of a polyketide synthase and a Nor-1-like ketoreductase, suggesting activation of pigment-associated secondary metabolism that may contribute to melanization and protection against PET-associated oxidative stress.

The induction of genes encoding a peroxidase, cytochrome P450, dienelactone hydrolase, aromatic ring-opening dioxygenase, and other oxidoreductases suggests that *A. niger* responds to PET by activating oxidative and xenobiotic metabolic pathways. Together with the upregulation of genes involved in adhesion, membrane transport, and oxidative stress protection, these findings support a model in which PET exposure triggers coordinated physiological adaptation to the polymer surface and facilitates the transformation of PET-associated compounds. While these genes represent promising candidates for PET-associated metabolism, their direct involvement in PET depolymerization remains to be experimentally validated.

In conclusion, this study links early marine fungal community structure with strain-level responses to pristine and weathered PET. Culture-based methods recovered a distinct, *Aspergillus*-enriched fraction of the fungal community and identified *A. niger* OC26 as a responsive model for studying fungal-plastic interactions. The candidate functional genes and physiological markers identified in this strain provide a foundation for screening proteins and enzymes involved in plastic surface attachment and modification. These findings may support the development of biotechnological tools for monitoring plastic biodeterioration and advancing fungal-based plastic treatment strategies.

## 4. Materials and Methods

### 4.1. Deployment of Plastic Pellets in the EMS

Sterilized industrial pellets of four polymers-PE (Ipethene^®^, Carmel Olefines, Haifa, Israel), PP (CAPILENE^®^ W 77 AV, Carmel Olefines, Haifa, Israel), PS (Styrolution^®^ PS 124L, INEOS Styrolution, Frankfurt, Germany), and PET (CZ-328, ZADE, Jiangyin, China) were deployed in the waters of the Herzliya Marina (32°09′38.8″ N 34°47′35.0″ E) during October 2024. Sterilized glass beads (5 mm, Cat. No. 104017, Sigma-Aldrich, St. Louis, MO, USA) served as non-plastic controls. Pellets were placed in sterile 5 × 5 cm organza mesh bags (MO45600, Apath international, Atlanta, Georgia) with six replicate bags per polymer (*n* = 24 total). All bags were submerged for 14 days at depths of 30–50 cm, retrieved, and rinsed with sterile, filtered artificial seawater (FASW) to remove loosely attached material.

### 4.2. Isolation of Pure Fungal Strains from Plastic and Stock Preparation

Fungal isolation was performed by following a modified protocol based on [50]. Five pellets from each mesh bag were placed onto 0.02% Chloramphenicol-supplemented isolation medium (Table S2). Emerging colonies were sub-cultured (2–3 transfers) onto fresh Malt Extract Agar (MEA) culturing plates (Table S3) until pure cultures were obtained. Purity was confirmed by consistent colony morphology and microscopic examination of colony structures, hyphae and fruiting bodies. For stock preparation, hyphae from actively growing colony margins were transferred into 2 mL cryotubes containing storage medium (Table S4) and incubated for one week. The cryotubes were then gradually frozen (2 h at 4 °C, 2 h at −20 °C and −80 °C for storage).

### 4.3. Microscopy and Morphological Characterization

Hyphae and fungal reproductive structures were visualized using lactophenol cotton blue (LPCB) staining. Sterile coverslips were placed on inoculated agar plates and incubated for 3–7 days. Colonized coverslips were then mounted on glass slides with LPCB solution (Sigma-Aldrich, St. Louis, MO, USA), gently washed to remove excess stain, and examined using a Nikon Eclipse Ci-L microscope (Nikon Corporation, Tokyo, Japan) equipped with a Nikon DS-Fi3 CMOS camera (Nikon Corporation, Tokyo, Japan).

### 4.4. DNA Extraction

DNA from environmental samples and pure fungal cultures was extracted using a phenol–chloroform-based protocol. Plastic pellets or fungal mycelium were transferred into lysis buffer (Table S5), together with approximately 0.4 g of sterile 425–600 µm glass beads. Samples were homogenized by bead beating and subjected to two freeze–thaw cycles in liquid nitrogen. Proteinase K (5 U/µL) was then added, and samples were incubated for 60 min at 55 °C until complete fungal tissue lysis was achieved. Subsequent extraction steps were performed according to the protocol described in [51].

### 4.5. ITS Barcode Amplification and Sequencing

The ITS2 region was amplified using primers ITS86-F (5′-GTGAATCATCGAATCTTTGAA-3′) and ITS4-R (5′-TCCTCCGCTTATTGATATGC-3′) as described in [52]. For isolate identification, PCR products were sequenced by Sanger (Hylabs Ltd., Rehovot, Israel). For community profiling, amplicons were purified (QIAquick PCR purification kit, QIAGEN, Hilden, Germany), quantified, and prepared using the Oxford Nanopore Technologies (Oxford Nanopore Technologies, Oxford, UK) Native Barcoding Kit (SQK-NBD114.24). Libraries were loaded and sequenced on a MinION Mk1B with an R10.4.1 flow cell (Oxford Nanopore Technologies, Oxford, UK).

### 4.6. Downstream ITS Read Processing and Analysis

To determine the taxonomic identity of individual fungal isolates, Sanger-derived ITS sequences were queried using BLASTn (v2.17.0) against the NCBI Core Nucleotide database (core_nt), and taxonomy was assigned based on the closest matches. To characterize fungal community structure, raw Nanopore reads (~24.7 million) were basecalled and trimmed using MinKNOW (v26.01.15) and subsequently processed with EPI2ME wf-16s v1.5.0. Based on nanopore MinION read-length analysis (Figure S1a), reads ranging from 300 to 500 bp were filtered for downstream analysis. Filtered reads were classified using Kraken2 against the EPI2ME default NCBI 16S/18S/28S/ITS targeted-loci database, using k = 35 and a minimum of 10 matching k-mers. Low-abundance taxa represented by fewer than 10 reads across the dataset were removed. Following filtering, 14,957,004 of 15,761,417 reads (94.9%) were successfully assigned taxonomy. Relative abundance data were normalized by total-sum scaling. Alpha diversity, including richness and Shannon diversity, and beta diversity based on Bray–Curtis dissimilarity and principal coordinate analysis (PCoA), were calculated using MicrobiomeAnalyst (v2.0) [53]. Differences between treatments were assessed using PERMANOVA (v0.2.0).

### 4.7. Plastic Weathering

WPET was obtained based on PPET sheets (6 mm thickness; SKYPET BR, ResMarts, TX, USA) that were subjected to accelerated UVB weathering using a QUV accelerated weathering tester (Q-Lab, Westlake, OH, USA). The PPET sheets were exposed to UVB radiation at 280–315 nm and an intensity of 1.2 W m^−2^ nm^−1^ for 2–7 days, as previously described in [14].

### 4.8. Preparation of Spore Suspension for the Inoculation of the Cultures

Spore suspensions were generated by washing sporulating isolates that were grown at 28 °C on MEA plates with 5 mL FASW containing 0.01% Tween 20. The resulting suspensions were filtered through sterile cotton to remove hyphal fragments, and spore concentrations were determined using a hemocytometer and adjusted to 1 × 10^6^ spores/mL with sterile FASW. Flasks were inoculated with 100 µL of the standardized spore suspensions.

### 4.9. Gravimetric PET Degradation Assay

PET degradation was assessed using a gravimetric approach. Five PPET and five WPET squares (1 × 1 cm) were prepared for each culture. The PET squares were weighed using high-precision analytic scale, sterilized with 70% ethanol and transferred to 250 mL flasks containing 50 mL of malt extract broth for the first week which was replaced with carbon-free Bushnell-Haas (BH) [14] after 7 days. Flasks were inoculated and incubated for 42 days at 28 °C with shaking at 80 rpm. Non-inoculated control flasks were included to monitor potential contamination and abiotic plastic mass changes. Following incubation, fungal hyphae were removed from the plastic using 1% sodium hypochlorite. The naked PET pieces were then rinsed with water and 70% ethanol, dried overnight, and reweighed. PET mass loss was calculated as the percentage reduction in mass relative to the initial weight: [(initial weight − final weight)/initial weight] × 100.

### 4.10. Peroxidase Activity Assay

To assess PET-induced peroxidase activity, isolates were inoculated using 100 µL of a spore suspension into 50 mL flasks containing approximately 1 g of UV-weathered PET sheets in malt extract broth. Cultures were incubated for one week at 28 °C with shaking at 80 rpm. Following incubation, the culture medium was collected and centrifuged at 20,000× *g* for 2 min to remove cellular debris.

For each sample, the supernatant was divided into two aliquots. One aliquot was heat-treated at 90 °C for 15 min to inactivate peroxidase activity, serving as background control, while the second aliquot remained untreated. Both aliquots were then loaded onto a 96-well plate in triplicate. Peroxidase activity in the untreated supernatant was quantified relative to the corresponding heat-treated control using the Amplex™ Red Hydrogen Peroxide/Peroxidase Assay Kit (Invitrogen, Thermo Fisher Scientific, Carlsbad, CA, USA), according to the manufacturer’s instructions. Fluorescence at 590 nm was measured using an Infinite 200 PRO microplate reader (Tecan Group Ltd., Männedorf, Switzerland).

### 4.11. Transcritpomic Response of A. niger Isolate OC26 to PET—Experimetal Setup

Isolate OC26 was cultured in the presence of PPET, WPET, or without plastic (NP). Cultures were grown in 250 mL flasks containing 50 mL malt extract broth with five PET sheets (1 × 1 cm) per flask, inoculated with 1 × 10^6^ spores/mL, and incubated in a shaker incubator at 28 °C, 80 rpm for 5–7 days. Mycelia were then harvested for RNA extraction as described below.

### 4.12. RNA Extraction

Collected mycelia were washed with double-distilled water (DDW), partially dried, and transferred into 2 mL cryotubes containing RNA-free glass beads (Sigma-Aldrich, St. Louis, MO, USA). Samples were flash-frozen in liquid nitrogen and lysed in DCT buffer supplemented with β-mercaptoethanol (1:100) using the InviTrap^®^ Spin Plant RNA Mini Kit (Invitek Molecular, Berlin, Germany). Tubes were incubated at 56 °C with intermittent vortexing and subjected to repeated snap freeze–thaw cycles until complete tissue disruption was achieved. RNA extraction was then completed according to the manufacturer’s protocol. RNA quality and concentration were assessed using an Agilent 4200 TapeStation (Agilent Technologies, Waldbronn, Germany).

### 4.13. cDNA Libraries Preperation and Sequencing

cDNA libraries were prepared using the ONT PCR-cDNA barcoding kit (PCB-SQK-111.24) (Oxford Nanopore Technologies, Oxford, UK) and sequenced on a MinION platform using a FLO-MIN106D (R9) (Oxford Nanopore Technologies, Oxford, UK) flow cell, generating ~12.78 Gb of data (~11.22 million reads). Basecalling, barcode assignment, and adapter trimming were performed using MinKNOW.

### 4.14. Transciptome Read Processing and Analysis

High-quality reads were aligned to the *A. niger* CBS 513.88 reference genome (GCF_000002855.4) using Minimap2 (v2.31) [54]. Gene counts were generated from BAM files using HTSeq [55] within the Galaxy platform (v26.0) [56] and imported into RStudio (v2026.07.1) [57] for downstream analysis. Genes with fewer than 0.5 counts per million (CPM) were filtered out, leaving 9354 genes for downstream analysis. Among these, 7795 genes were successfully converted to Ensembl gene IDs, while the remaining 1559 genes were retained with their original identifiers. Differential gene expression analysis was performed using the iDEP.96 platform [58]. Data was normalized using regularized log transformation for principal component analysis and clustering. Differentially expressed genes were identified using DESeq2 [59], applying thresholds of fold change ≥ 2 and false discovery rate (FDR) ≤ 0.1. Functional enrichment analysis of differentially expressed genes was performed using DAVID [60], with the *A. niger* gene set used as the background. Intersections among gene sets were visualized using the UpSetR (v1.4.1) [61].

## Figures and Tables

**Table 1:** Taxonomic identity and occurrence frequency of fungal isolates from incubated samples.

Isolate	ITS-Based Taxonomic Identification	Phylum	Total Isolates (*n*)
OC2	*Cladosporium sphaerospermum*	Ascomycota	23
OC3	*Mucor* sp. *	Mucoromycota	5
OC6	*Aspergillus oryzae* **	Ascomycota	2
OC7	*Aspergillus nidulans*	Ascomycota	3
OC8	*Allophoma yuccae*	Ascomycota	6
OC9	*Alternaria destruens*	Ascomycota	11
OC10.2	*Aspergillus chevalieri*	Ascomycota	1
OC11	*Penicillium steckii*	Ascomycota	5
OC12	*Aspergillus oryzae* **	Ascomycota	3
OC13	*Purpureocillium lilacinum*	Ascomycota	1
OC14	*Aspergillus ochraceus*	Ascomycota	1
OC15	*Waltergamsia pilosa*	Ascomycota	8
OC18	*Aspergillus violaceus*	Ascomycota	2
OC19	*Candida parapsilosis*	Ascomycota	2
OC20	*Aspergillus sydowii*	Ascomycota	4
OC21	*Aspergillus latilabiatus*	Ascomycota	1
OC22	*Penicillium hirsutum*	Ascomycota	2
OC23	*Aspergillus baeticus*	Ascomycota	1
OC24	*Aspergillus terreus*	Ascomycota	1
OC25	*Cyphellophora oxyspora*	Ascomycota	1
OC26	*Aspergillus niger*	Ascomycota	26

* Based on morphology alone. ** Identified as different strains by morphology (Figure S4).

**Table 2:** Weight loss, pigmentation and peroxidase activity of *Aspergillus* isolates in the presence of WPET and PPET.

Isolate *	Weight Lost (WPET vs. PPET) **	Attachment		Pigmentation		Peroxidase Activity WPET (A 590 nm) Compared to Heat-Inactivated Control
		PPET	WPET	PPET	WPET	
OC6	-	-	-	++	++	0.67
OC7	-	-	+	++	++	0
OC10.2	0.15%	-	+	+++	+++	0
OC12	0.15%	+	+	++	+	1.67
OC14	0.17%	-	+	++++	+	7.67
OC20	0.1%	-	-	+++	+++	9.33
OC21	-	+	+	+++	+++	2
OC23	0.21%	-	-	n/a	++	n/a
OC24	0.17%	-	+	++	++	0
OC26	0.2%	-	+	+	++	758

* Isolate OC18 did not grow from stock. ** Average percent (*n* = 3). (-, no response; +, weak; ++, moderate; +++, strong; ++++, very strong; n/a, not determined.)

## Data Availability

All sequencing data generated in this study have been deposited in NCBI databases. *A. niger* isolate OC26 ITS barcode is available in GenBank under accession numbers PZ359197-PZ359216. Nanopore-based community ITS metabarcoding data and RNA-seq data are available in the NCBI Sequence Read Archive (SRA) under BioProject accession number PRJNA1467171 and PRJNA1455216, respectively.

## Supplementary Materials

The following supporting information can be downloaded at https://www.mdpi.com/article/10.3390/ijms27156861/s1.
