# Supplementary material for: Plastic-Colonizing Fungi in the Eastern Mediterranean Sea: Community Structure and Physiological and Transcriptional Responses of Aspergillus to Pristine and Weathered PET
Source: Int J Mol Sci. 2026 Jul 30;27(15):6861. doi: 10.3390/ijms27156861 (PMC13466381; doi:10.3390/ijms27156861)
Supplement: Supplementary file 1 [file ijms-27-06861-s001.zip › ijms-4468718-supplementary.pdf]

Plastic-Colonizing Fungi in the Eastern Mediterranean Sea: Community Structure and Physiological and Transcriptional Responses of *Aspergillus* to Pristine and Weathered PET

Sheli Shlomo Itzahri, Denis Popov, Keren Davidov and Matan Oren*

Department of Molecular Biology, Ariel University, Science Park, Ariel 40700, Israel.
* Correspondence: matanor@ariel.ac.il

Supplementary Materials

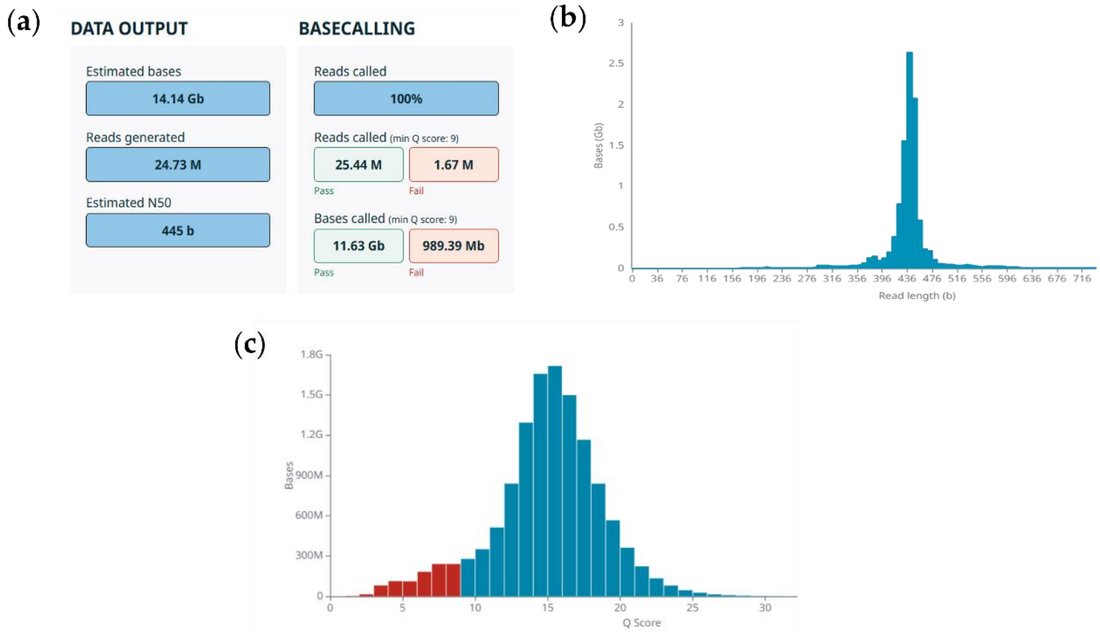

Figure S1. Nanopore MinION run report. (**a**) Data output of the sequencing run. (**b**) Distribution of the ITS reads according to length (before trimming). (**c**) Reads' quality score, with a cutoff of Q score ≤ 9.

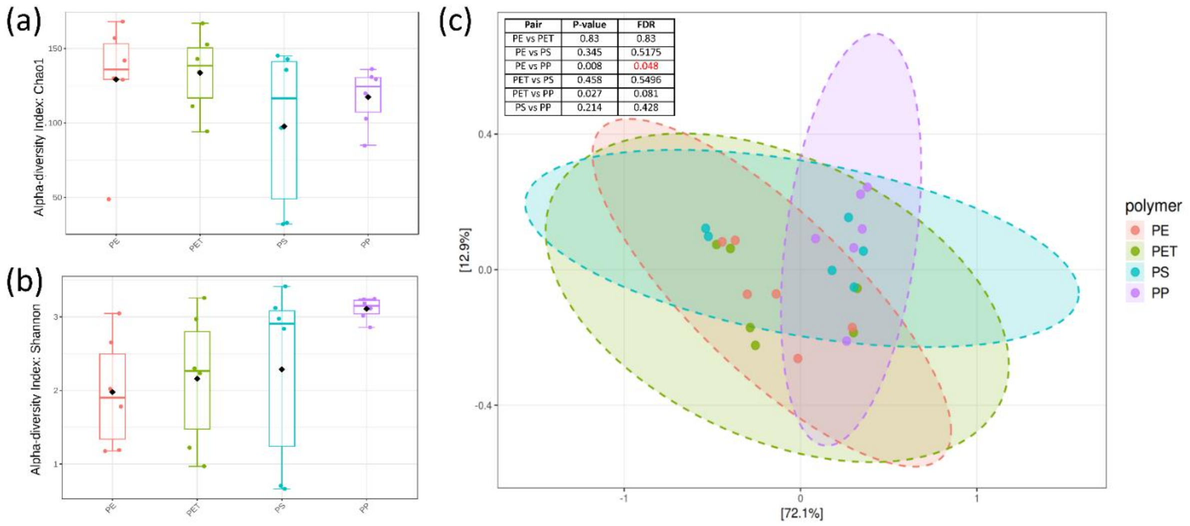

Figure S2. Taxonomic diversity of plastic-associated fungal communities grouped by polymer type (PE, PET, PP, PS). (**a**) Taxon richness is estimated by Chao1 index. (**b**) Community diversity estimated by Shannon index (Kruskal–Wallis test, p = 0.027). (**c**) PCA beta diversity analysis for dissimilarity among samples (PERMANOVA test values are presented in the table; significant FDR value is emphasized in red).

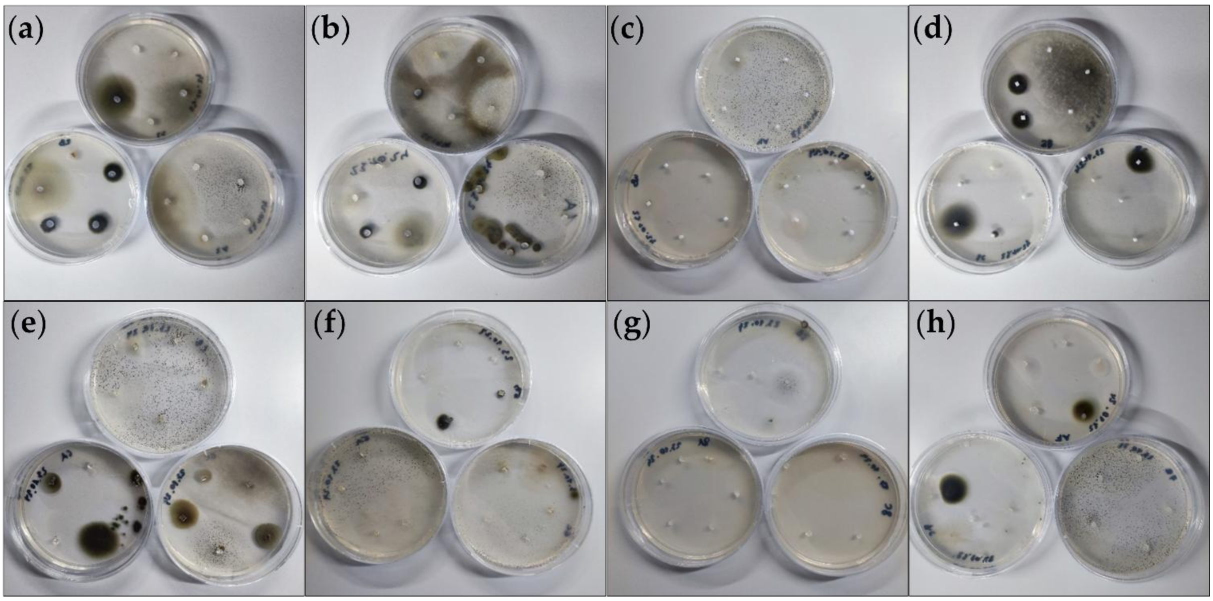

Figure S3. Fungal growth one week after inoculating plastic pellets retrieved from the Herzliya Marina onto solid malt-extract agar plates. (**a-b**) PE pellets. (**c-d**) PET pellets. (**e-f**) PS pellets. (**g-h**) PP pellets.

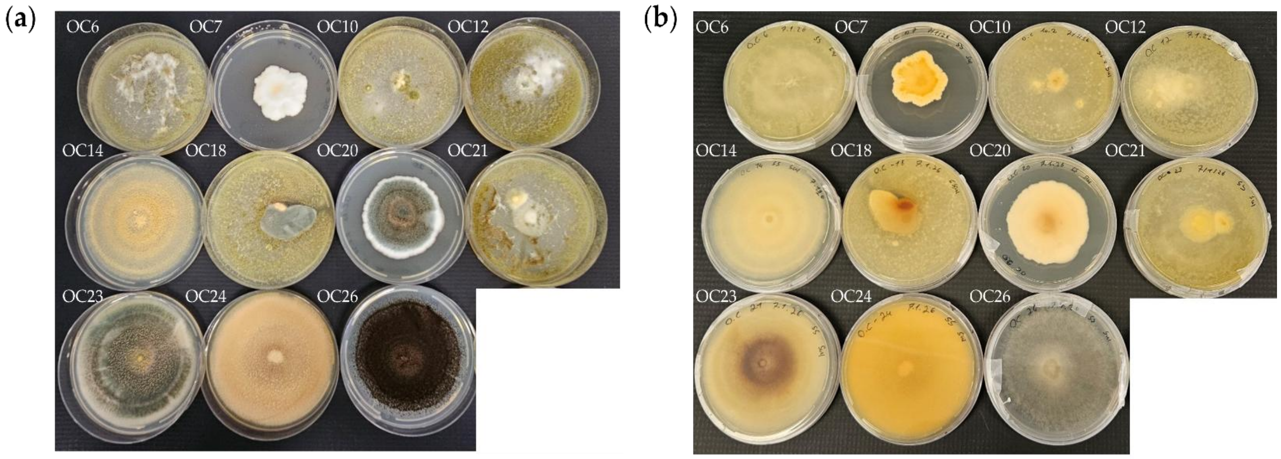

Figure S4. Pure cultures of plastic-derived *Aspergillus* isolates on Malt Extract Agar (MEA) plates: (**a**) view from above, (**b**) view from below.

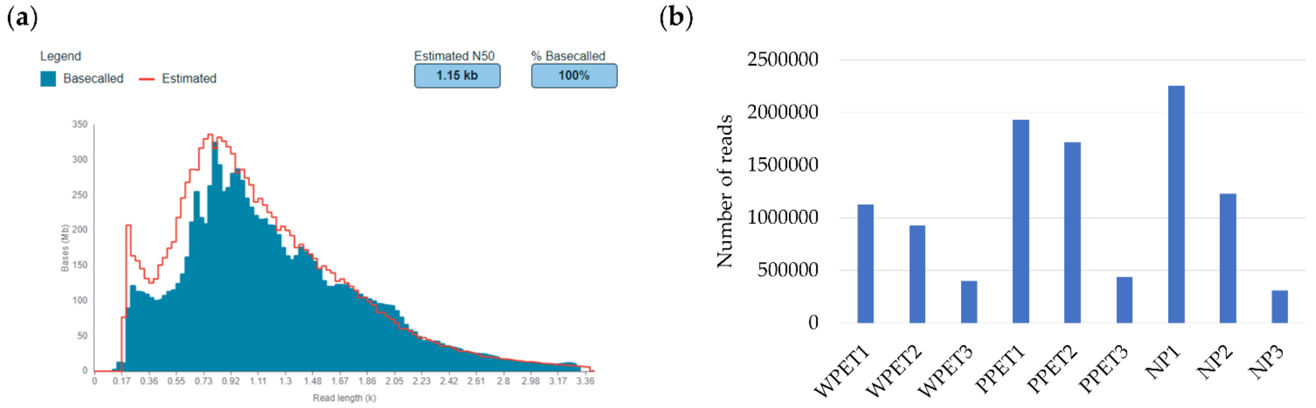

Figure S5. MinION Mk1B run report. (**a**) total number of bases vs the read length. (**b**) Number of reads per barcode sequenced.

Table S1. Distribution of morphologically distinct fungal isolates across inoculated samples according to substrate type

Isolate	ITS-based taxonomic identification	Colony color	Pellets yielding isolate (n)					Total (n)
			PE (30)	PP (30)	PS (30)	PET (30)	Glass (9)	
OC2	*Cladosporium sphaerospermum*	Greenish brown	8	3	6	6	0	23
OC3	*Mucor sp.*[1]	Brown/gray	2	0	0	3	0	5
OC6	*Aspergillus oryzae*	Swamp green	1	0	1	0	0	2
OC7	*Aspergillus nidulans*	Yellow/Green	1	1	1	0	0	3
OC8	*Allophoma yuccae*	Brown	6	0	0	0	0	6
OC9	*Alternaria destruens*	Grey	5	0	3	3	0	11
OC10.2	*Aspergillus chevalieri*	Greenish yellow	0	0	0	1	0	1
OC11	*Penicillium steckii*	Sea green	0	2	2	1	0	5
OC12	*Aspergillus oryzae*	Green	0	0	2	1	0	3
OC13	*Purpureocillium lilacinum*	Pale pink	0	0	0	1	0	1
OC14	*Aspergillus ochraceus*	Brownish yellow	0	0	1	0	0	1
OC15	*Waltergamsia pilosa*	Grey, pale pink center	1	6	0	0	1	8
OC18	*Aspergillus violaceus*	Sea green	0	2	0	0	0	2
OC19	*Candida parapsilosis*	White	0	2	0	0	0	2
OC20	*Aspergillus sydowii*	Sea green	0	1	3	0	0	4
OC21	*Aspergillus latilabiatus*	Green	0	1	0	0	0	1
OC22	*Penicillium hirsutum*	Sea green	0	1	0	1	0	2
OC23	*Aspergillus baeticus*	White	0	1	0	0	0	1
OC24	*Aspergillus terreus*	Pale pink	0	1	0	0	0	1
OC25	*Cyphellophora oxyspora*	Gray/green	0	0	0	0	1	1
OC26	*Aspergillus niger*	Black	5	5	12	4	0	26

[1] based on morphology.

Table S2. Isolation medium

Name	Manufacturer	Catalog number	Amount for 1L media
Bacto agar	LLG-Labware	BCCC5380	15g
Malt extract	Gibco	218630	15g
Chloramphenicol	Arcos organics	227920250	0.2g
Red Sea Salt Mix	Red Sea	R11065	24.4g
DDW (double distilled water)	-	-	Fill to 1000 ml

Table S3. Malt Extract Agar (MEA) culturing medium

Name	Manufacturer	Catalog number	Amount for 1L media
Bacto agar	LLG-Labware	BCCC5380	15g
Malt extract	Gibco	218630	15g
Red Sea Salt Mix	Red Sea	R11065	24.4g

DDW (double distilled water)	-	-	Fill to 1000 ml

Table S4. Storage medium

Name	Manufacturer	Catalog number	Amount for 1L media
Bacto agar	LLG-Labware	BCCC5380	15g
Malt extract	Gibco	218630	15g
Glycerol	Carlo erba	453752	50g
Yeast extract	LLG-Labware	6271004	0.1g
Red Sea Salt Mix	Red Sea	R11065	24.4g
DDW (double distilled water)	-	-	Fill to 1000 ml

Table S5. Lysis buffer

Name	Stock concentration	Final concentration in buffer	Amount for 1 L buffer
Tris-HCl (pH 8.0)	1 M	10 mM	10 mL
Na_2EDTA (pH 8.0)	0.5 M	25 mM	50 mL
SDS	10% w/v	1% (w/v)	100 mL
NaCl	5 M	100 mM	20 mL
DDW (double distilled water)	–	–	Fill to 1000 mL

Table S6. List of differentially expressed genes in sample groups of interest

Description	Gene Name	PPET-NP_log2FC	FDR	WPET-NP_log2FC	FDR
**MFS transporter**	An16g01000	1.69	0.005105	3.65	4.37E-13
**hypothetical protein**	An16g01010	1.77	0.061338	4.62	3.03E-12
**salicylate hydroxylase**	An07g00550	1.14	0.141828	1.61	0.020285
**hypothetical protein**	An01g05810	1.21	3.33E-06	1.49	2.60E-08
**DNA binding protein SART-1**	An04g06370	0.89	0.017022	1.45	1.50E-05
**trehalose synthase (Ccg-9)**	An02g07770	1.26	0.012297	1.28	0.01556
**hypothetical protein**	An11g01830	1.30	0.171286	1.33	0.181844
**hypothetical protein**	An04g06580	2.52	0.02264	2.25	0.101774
**formamidase**	An07g05830	1.45	0.054202	3.67	2.63E-11
**hypothetical protein**	An02g12650	1.76	4.30E-06	1.71	4.89E-05
**DNA repair protein Rhp26/Rad26**	An14g01060	2.00	0.015869	1.89	0.058416
**protein kinase (Chm1)**	An17g02010	4.34	0.006928	1.87	0.648372
**haloacid dehalogenase-like hydrolase**	An07g05760	1.59	0.163743	2.23	0.023698
**2-amino-3-carboxymuconate-6-semialdehyde decarboxylase**	An02g08220	1.38	5.32E-11	1.52	3.31E-12
**hypothetical protein**	An08g00540	1.18	6.01E-05	1.51	1.26E-07
**nitrite reductase [NAD(P)H]**	An08g05640	2.30	0.033219	3.85	2.16E-05
**aspartic endopeptidase (AP1)**	An01g00370	1.28	0.001651	2.27	4.92E-10
**nitrate transporter**	An08g05670	2.53	0.002675	3.86	4.08E-07
**hypothetical protein**	An07g06170	1.16	2.91E-06	1.67	1.07E-11

**hypothetical protein**	An02g12940	1.54	0.148737	1.72	0.122641
**methionine aminopeptidase 1**	An01g11340	5.37	0.002505	3.27	0.289757
**dienelactone hydrolase**	An14g01430	2.13	3.36E-06	2.10	3.68E-05
**pathogenesis associated protein Pep2**	An02g03200	1.33	8.04E-07	1.60	1.30E-08
**hypothetical protein**	An09g02830	1.85	0.239271	2.77	0.025872
**hypothetical protein**	An07g05030	1.11	0.603983	1.71	0.252659
**proline-specific permease**	An11g06150	1.18	0.189343	1.69	0.019666
**cysteine protease atg4**	An11g11320	2.76	1.13E-09	1.18	0.153041
**hypothetical protein**	An04g05970	1.58	0.434026	1.84	0.340859
**hypothetical protein**	An03g02530	1.73	0.042112	2.25	0.00591
**protein PRO41**	An08g10010	2.02	0.023633	2.43	0.00679
**hypothetical protein**	An14g05570	1.72	0.232963	2.07	0.129699
**hypothetical protein**	An09g02920	1.59	1.25E-05	2.10	2.95E-09
**cell polarity protein (Alp11)**	An07g10210	5.43	1.16E-05	1.82	0.627614
**hypothetical protein**	An03g02780	4.77	0.013247	2.88	0.434054
**alcohol dehydrogenase 2**	An17g01530	1.17	0.004007	1.44	0.000193
**hypothetical protein**	An02g07440	3.13	0.214741	3.50	0.15121
**hypothetical protein**	An01g02760	2.36	4.47E-07	2.18	2.52E-05
**UDP-glucoronosyl and UDP-glucosyl transferase**	An16g05970	1.20	0.022822	1.67	0.000578
**hypothetical protein**	An17g01760	0.94	0.529851	1.39	0.210801
**hypothetical protein**	An07g05420	1.95	0.157573	2.95	0.008489
**O-methyltransferase**	An14g05840	2.19	0.167063	2.24	0.197971
**hypothetical protein**	An16g07420	1.11	0.010733	1.35	0.001599
**catalase R**	An01g01550	3.80	0.198549	3.18	
**FAS1 domain-containing protein**	An16g07370	1.92	0.000329	0.98	0.304674
**NAD dependent epimerase/dehydratase**	An16g02240	2.06	0.056893	2.54	0.015865
**hypothetical protein**	An03g04400	1.72	0.000114	2.36	4.03E-08
**hypothetical protein**	An16g02200	7.81	3.24E-10	2.06	0.607275
**hypothetical protein**	An18g05790	0.93	0.283126	1.33	0.063412
**oxidation resistance protein 1**	An16g02160	2.00	0.004976	1.79	0.035811
**hypothetical protein**	An02g03980	1.64	0.004	2.02	0.000394
**hypothetical protein**	An01g07090	4.70	0.067503	3.70	
**hypothetical protein**	An08g06620	0.91	0.180656	2.16	4.25E-06
**HAD superfamily hydrolase**	An03g04530	1.14	0.000823	1.11	0.002398
**Ser/Thr protein phosphatase family protein**	An01g03170	4.12	0.015423	2.40	0.466848
**tRNA a64-2'-o-ribosylphosphate transferase**	An03g04550	3.00	0.000446	1.81	0.210801
**hypothetical protein**	An08g01700	1.16	0.000321	2.11	1.65E-12
**integral membrane protein**	An13g01340	3.29		2.84	
**hypothetical protein**	An03g04430	1.16	0.156569	1.41	0.086668
**acetamidase**	An11g02980	4.80	1.25E-09	2.10	0.161561
**hypothetical protein**	An18g00910	2.99	1.81E-06	2.14	0.009781
**aromatic amino acid aminotransferase**	An09g05080	1.42	0.167063	1.98	0.029037
**polysaccharide deacetylase**	An15g00280	2.15	0.041681	3.76	1.22E-05

**oxidoreductase, short chain dehydrogenase/reductase family**	An04g08020	1.33	0.10888	1.43	0.117242
**HLH DNA binding domain protein**	An14g02540	1.39	0.000842	1.69	3.42E-05
**hypothetical protein**	An08g01870	2.40	5.81E-22	1.17	0.000518
**non-classical export protein Nce2**	An03g04860	1.17	0.000963	1.37	0.000125
**Rad21/Rec8 N terminal domain protein**	An12g07590	2.64	0.460443	3.08	
**MFS multidrug transporter**	An04g08250	1.21	0.409406	1.75	0.110033
**hypothetical protein**	An18g06380	1.13	0.15205	1.45	0.049557
**phosphoketolase**	An13g01680	0.72	0.448581	1.86	0.000355
**hypothetical protein**	An03g04770	1.36	0.057744	1.22	0.183158
**aromatic ring-opening dioxygenase LigB subunit**	An12g07530	2.44	2.24E-07	1.53	0.020601
**hypothetical protein**	An08g12250	1.38	0.013087	1.74	0.001058
**hypothetical protein**	An01g00610	1.40	0.000898	2.17	2.74E-08
**inositol-pentakisphosphate 2-kinase**	An04g07130	1.60	0.001779	1.89	0.000327
**O-methyltransferase**	An01g15020	2.46	0.217328	3.42	0.035774
**DNA repair protein rad14**	An03g03720	5.71	7.33E-06	3.02	0.168217
**MFS multidrug transporter**	An07g01220	2.25	0.269577	2.96	0.082816
**D-lactate dehydrogenase**	An01g09780	1.26	0.10127	2.18	0.000358
**mitotic spindle checkpoint component mad2**	An09g04120	6.86	2.23E-05	2.91	0.452494
**nitrilase**	An12g01260	1.12	0.104102	2.23	2.12E-05
**nucleoside transporter**	An04g07070	0.99	0.00255	1.02	0.003079
**hypothetical protein**	An07g06280	1.53	0.085352	2.29	0.003079
**hypothetical protein**	An11g02190	2.91	0.107531	4.45	0.00214
**hypothetical protein**	An14g06630	1.73	0.027274	2.43	0.000893
**hypothetical protein**	An08g11120	4.26		2.86	
**endopolygalacturonase C**	An01g14670	2.10	0.002441	2.79	4.46E-05
**aflatoxin biosynthesis ketoreductase nor-1**	An11g02460	1.16	0.078197	1.47	0.02301
**hypothetical protein**	An01g07120	1.02	0.141828	2.77	2.85E-09
**hypothetical protein**	An11g07720	2.83	0.115445	2.70	0.194026
**carboxylic acid transport protein**	An16g06950	2.05	0.150136	1.89	0.282006
**integral membrane protein**	An11g02490	4.31	0.050185	3.54	0.223737
**opsin-1**	An12g01460	2.46	8.61E-08	3.03	8.03E-11
**D-xylulose reductase A**	An07g01290	1.19	0.010097	1.07	0.03089
**metallo-beta-lactamase superfamily protein**	An04g07220	1.96	0.00013	2.53	1.04E-06
**hypothetical protein**	An16g06810	1.22	0.021409	1.74	0.000302
**alpha-ketoglutarate-dependent taurine dioxygenase**	An16g01950	2.02	0.008336	2.63	0.000442
**hypothetical protein**	An02g08810	1.31	0.058803	1.29	0.106173
**hypothetical protein**	An15g04670	3.18	0.009246	3.72	0.002247
**amino-acid permease inda1**	An13g00840	1.45	0.008534	2.70	1.11E-08
**RPEL repeat protein**	An12g01540	0.71	0.38601	1.02	0.083907
**cortical patch protein SUR7**	An07g06530	2.17	1.72E-06	2.66	1.07E-08
**geranylgeranyl diphosphate synthase**	An11g02500	3.36	0.200713	3.19	0.282965

**hypothetical protein**	An18g00390	1.30	0.064756	1.95	0.001599
**zinc alcohol dehydrogenase**	An11g07750	2.59	0.001086	1.64	0.211561
**BYS1 domain protein**	An16g01850	1.36	0.028498	1.85	0.001277
**zinc-regulated transporter 1**	An01g01620	3.32	0.141352	4.40	0.023286
**hypothetical protein**	An05g01880	2.12	0.090772	2.15	0.127047
**fructosyl amine:oxygen oxidoreductase**	An02g08980	1.46	0.163743	2.77	0.000328
**MFS transporter**	An02g08970	1.37	0.078621	3.40	9.02E-10
**C2H2 finger domain protein**	An09g04540	0.97	0.090681	1.26	0.02156
**kinesin light chain**	An08g11610	2.22	0.39316	2.89	0.17158
**hypothetical protein**	An04g07500	1.68	0.340373	1.81	0.301431
**hypothetical protein**	An16g03500	1.32	3.01E-05	1.74	1.20E-08
**integral membrane protein**	An01g01475	1.25	0.141828	1.83	0.013761
**hypothetical protein**	An16g08670	1.41	0.002094	1.50	0.001786
**hypothetical protein**	An01g14000	2.82	0.095033	3.71	0.015565
**MYND-type zinc finger protein samB**	An18g02060	1.67	3.67E-06	1.40	0.000845
**acetylserotonin methytransferase-like protein (ASMTL)**	An01g13940	3.97	0.000232	2.02	0.344073
**inulinase**	An12g08280	1.33	0.002978	1.30	0.01076
**hypothetical protein**	An16g03720	2.76	1.16E-10	1.83	0.000689
**proteinase**	An12g08560	1.18	0.084243	1.91	0.00103
**hypothetical protein**	An01g09900	1.65	0.023305	3.20	7.26E-08
**peroxidase**	An02g10480	1.59	0.069243	1.79	0.053859
**oxidoreductase, zinc-binding dehydrogenase family**	An09g01080	1.77	0.022074	2.32	0.00214
**Zn(II)2Cys6 transcription factor**	An11g09590	1.34	0.073648	1.42	0.071727
**aspartic protease pepAc**	An12g03300	1.59	0.000158	1.65	0.000471
**hypothetical protein**	An06g00720	3.61	0.031187	3.21	0.10897
**transmembrane amino acid transporter family protein**	An03g00640	0.99	0.147765	1.66	0.001879
**pyruvate dehydrogenase**	An02g00580	2.61	0.369113	2.19	
**hypothetical protein**	An01g03530	1.18	1.26E-05	0.92	0.004918
**hypothetical protein**	An02g05620	1.15	0.000134	2.01	4.60E-13
**IQ calmodulin-binding motif protein**	An02g05600	2.84	0.507457	2.38	
**oxidoreductase, short chain dehydrogenase/reductase family**	An04g04170	1.98	0.002896	1.99	0.005126
**MFS allantoate transporter**	An12g03550	1.63	0.228304	4.09	7.69E-07
**ammonium transporter 1**	An08g03200	1.39	0.076414	1.52	0.044481
**hypothetical protein**	An02g05640	1.00	0.038062	2.09	5.94E-08
**potassium transporter**	An02g05630	1.06	0.021064	2.19	6.80E-09
**hypothetical protein**	An09g01230	6.85	2.80E-20	2.70	0.027394
**short chain oxidoreductase/dehydrogenase**	An02g10680	3.25	0.009284	2.53	0.136517
**hypothetical protein**	An13g02730	4.23	0.011681	6.74	2.43E-06
**hypothetical protein**	An08g03390	1.12	0.007624	1.46	0.000273
**polyketide synthase**	An04g04340	3.43	0.018784	3.09	0.072464
**hypothetical protein**	An16g09160	3.21	9.70E-07	1.94	0.048839

**cellulase family protein**	An03g01050	3.86	0.246021	4.93	
**ceramide very long chain fatty acid hydroxylase SCS7**	An01g14200	1.24	0.000869	1.16	0.003479
**isoflavone reductase family protein**	An07g03520	1.51	0.329084	1.25	0.460403
**C-5 sterol desaturase**	An16g02930	1.54	3.08E-08	1.10	0.000728
**cytochrome P450**	An11g08810	3.69	0.235565	3.55	
**mannose-6-phosphate isomerase, class I**	An04g03200	1.74	0.026745	2.12	0.006071
**hypothetical protein**	An18g06410	1.29	0.000734	1.50	0.000232
**hypothetical protein**	An14g03080	1.29	2.94E-06	1.64	1.65E-09
**proteasome component C5**	An18g06700	5.13	3.51E-30	1.72	0.02467
**hypothetical protein**	An09g05625	1.24	2.93E-11	1.23	3.57E-10
**hypothetical protein**	An12g07920	1.25	7.80E-05	2.19	2.51E-13
**hypothetical protein**	An01g03130	1.49	0.000378	1.49	0.000633
**hypothetical protein**	An16g08140	1.80	0.113669	1.60	0.282868
**hypothetical protein**	An08g02380	1.21	0.002392	1.42	0.000542
**hypothetical protein**	An09g00320	2.37	0.404865	2.60	
**hypothetical protein**	An01g08840	2.47	0.158396	2.84	0.099857
**sphinganine hydroxylase BasA**	An01g10030	1.88	3.60E-11	2.17	1.04E-13
**aorsin**	An01g01750	1.87	0.064457	4.58	9.78E-11
**hypothetical protein**	An10g00160	3.44	0.010824	3.40	0.023467
**hypothetical protein**	An18g01790	1.08	0.312863	1.64	0.046909
**hypothetical protein**	An18g06740	5.79	6.22E-07	3.75	0.016659
**hypothetical protein**	An01g13470	1.42	0.184718	1.72	0.097661
**MFS multidrug transporter**	An01g04100	4.30	0.088932	2.05	
**hypothetical protein**	An15g07560	1.18	0.000121	1.43	5.28E-06
**hypothetical protein**	An08g04120	1.08	0.098565	1.48	0.009635
**hydantoinase/oxoprolinase**	An02g00170	2.76	0.009252	2.28	0.105589
**F1F0-ATP synthase regulatory factor Stf2**	An04g00010	1.06	0.056316	1.43	0.003467
**phosphoglycerate mutase family protein**	An15g07520	1.35	0.069126	1.17	0.235879
**GPI mannosyltransferase 2**	An04g05330	0.93	0.071412	0.96	0.087091
**RING finger domain protein**	An14g04970	2.63	8.42E-08	1.75	0.008698
**hypothetical protein**	An09g02320	4.88	7.01E-09	1.79	0.374359
**hypothetical protein**	An07g04500	1.04	0.070764	1.13	0.06516
**oxaloacetate hydrolase class protein**	An15g07720	1.06	0.163584	1.31	0.07353
**hypothetical protein**	An09g02330	8.02	2.24E-08	2.89	0.42839
**aconitase family protein**	An15g07730	1.13	0.218602	1.32	0.164486
**allergen Asp f 15**	An02g01550	1.12	0.035213	1.29	0.013761
**adhesin**	An16g04900	3.63	0.00456	1.96	0.453247
**glutathione S-transferase**	An07g09590	0.91	0.136819	1.36	0.007536
**spindle poison sensitivity protein Scp3**	An11g11070	1.02	0.026856	1.37	0.002122
**hypothetical protein**	An12g04880	0.96	0.089568	1.27	0.011034
**serine peptidase**	An08g04490	1.07	0.065083	2.55	4.36E-09
**L-asparaginase**	An01g14960	1.60	0.026856	2.45	0.000102
**THUMP domain protein**	An02g06810	1.01	0.004945	1.19	0.000877
**hypothetical protein**	An01g11320	1.89	9.94E-05	3.98	6.85E-21

**hypothetical protein**	An06g02170	2.61	0.27705	3.51	0.073893
**pyruvate decarboxylase**	An02g06820	0.93	0.154963	1.37	0.008529
**integral membrane protein**	An07g04900	1.38	2.66E-09	1.74	2.38E-13
**DnaJ domain protein**	An18g03870	1.00	0.010926	1.01	0.016126
**integral membrane protein**	An01g11940	2.11	0.186067	1.99	0.308934
**RNA export mediator Gle1**	An08g03550	0.97	0.184718	0.99	0.226022
**hypothetical protein**	An08g03560	1.29	0.353723	1.63	0.14139
**hypothetical protein**	An11g10070	2.79	0.013638	1.59	0.454546
**hypothetical protein**	An11g04830	1.21	0.215389	1.72	0.043096
**hypothetical protein**	An13g03080	1.71	0.005207	1.36	0.077313
**hypothetical protein**	An09g06610	0.98	0.249778	1.42	0.047666
**mannitol-1-phosphate 5-dehydrogenase**	An02g05830	0.80	0.286888	0.88	0.193731
**hypothetical protein**	An06g01220	1.00	0.168314	1.24	0.07566
**hypothetical protein**	An15g02040	1.59	7.72E-05	2.14	2.49E-08
**hypothetical protein**	An12g04090	3.16	0.022862	4.62	0.000166
**hypothetical protein**	An08g03700	1.34	0.005024	1.17	0.038536
**protein kinase**	An18g02860	1.05	0.015721	1.93	2.21E-07
**O-methyltransferase**	An01g15010	1.93	0.069055	2.91	0.001303
**hypothetical protein**	An03g06500	1.72	0.322331	2.15	0.164426
**hypothetical protein**	An02g01040	1.89	0.225437	1.99	0.22379
**integral membrane protein**	An12g04070	1.11	0.025591	1.68	0.000212
**2,4-dichlorophenol 6-monooxygenase**	An04g04680	6.97	1.44E-05	3.61	0.240682
**hypothetical protein**	An02g00970	3.21	3.34E-19	1.26	0.028057
**ABC transporter**	An09g01700	2.93	0.021927	2.96	0.036878
**hypothetical protein**	An02g06060	1.33	0.050452	1.38	0.04785
**phosphoribosyl-aminoimidazole-succinocarboxamide synthase**	An11g10160	1.24	0.373219	1.40	0.32423
**translation initiation factor eIF-2B subunit family protein**	An02g00980	1.51	1.38E-07	1.36	8.11E-06
**C-4 methylsterol oxidase**	An03g06410	1.02	5.82E-07	1.01	2.97E-06
**MFS transporter**	An15g02190	1.02	0.265625	2.09	0.00074
**reductase**	An08g03830	1.76	0.187979	1.50	0.4114
**hypothetical protein**	An19g00210	1.26	0.000748	1.61	6.75E-06
**hypothetical protein**	An15g02180	2.57	0.080231	3.02	0.03089
**primary amine oxidase 2**	An17g00010	2.59	0.014434	5.34	2.83E-10
**hypothetical protein**	An15g07200	0.91	0.141828	0.79	0.301431
**HPP family protein**	An17g00230	1.54	0.022822	1.67	0.020117
**hypothetical protein**	An08g03760	1.07	0.000601	2.31	7.64E-17
**hypothetical protein**	An15g07220	2.29	1.25E-05	1.32	0.097115
**OPT oligopeptide transporter**	An11g05350	1.38	0.016661	3.46	4.75E-14
**phosphatidylglycerol specific phospholipase**	An13g03600	5.75	0.005333	2.87	
**sister chromatid cohesion acetyltransferase Eco1**	An11g10560	1.27	0.078375	1.94	0.001927
**acetate kinase**	An02g06420	2.43	6.19E-08	3.22	8.61E-13
**fructosyl amine:oxygen oxidoreductase**	An11g05340	1.80	0.000112	2.42	6.51E-08

**arginine permease**	An03g01590	1.62	0.090681	4.52	2.62E-12
**general amidase-B**	An08g09020	1.26	0.196512	1.52	0.099823
**vacuolar calcium ion transporter**	An19g00320	5.27	5.54E-05	2.38	0.412002
**dehydrase family protein**	An07g09240	4.43	0.032357	3.39	
**hypothetical protein**	An03g01630	2.59	1.89E-08	2.79	6.91E-09
**hypothetical protein**	An01g07060	1.29	0.000514	2.16	6.82E-10
**FAD binding domain protein**	An02g06340	0.82	0.381618	1.14	0.139058
